# Street Noises

**Published:** 1898-05-21

**Authors:** 


### Street Noises.

To all lovers of quietness, to all brain-workers, and to all haters of the vile noises by which so many cities are rendered hideous, the news will be welcome indeed that the Judges of the Queen's Bench Division have upheld the right of a county council to make and enforce bye-laws framed with the object of putting a stop to some, at least, of the noises of the streets.

The case itself was delightfully simple, although the legal problems it involved were somewhat complex. The Kent County Council made a bye-law prohibiting the playing of musical instruments and singing "in any public place or highway within fifty yards of any dwelling-house" after a request to desist either by the occupants or their servants or by a police-constable, and in regard to the case in question there was no dispute as to the facts. That the occupier of a house had repeatedly complained of the annoyance of street singing, and that the appellant had persisted in singing in a public highway within fifty yards of a dwelling-house, after having being required by a police-constable to desist, was all admitted, and on this the magistrate had convicted. The question reserved for the Court was, whether the bye-law under which the action was taken was valid; and this question hinged upon the further question whether such a bye-law was "reasonable." This was reviewed by the Lord Chief Justice in an elaborate and careful judgment, in which he first of all drew a broad distinction between bye-laws framed by private bodies—railway companies, for example—and bye-laws framed by representative bodies, such as municipal and county councils, which are elected by those who dwell in the districts within which these bye-laws apply, and where they have the force of law. The Lord Chief Justice argued that the latter should be supported by the Courts, unless they were obviously unreasonable, pointing out how complete were the safeguards by which the power of local bodies to make bye-laws had been hedged round by the Legislature, and how unlikely it was that any such bye-laws would be passed if serious objection were raised by those whom they would affect. This, in fact, is the keynote of what is reasonable, namely, that the bye-law shall express the intention of the local public. Thus the conviction was upheld.

The importance of this decision it is difficult to over-estimate. No doubt there are some coarse-nerved and leaden-fibred individuals who will pity the "poor organ grinder," and some, perhaps, so full of religious tolerance that, for the sake of the possible good done by the Salvation Army, they would have them beat their drums even on the day of rest; but there can be no doubt that the vast majority of the dwellers in towns, including the whole mass of those who work with their brains, and all the sick, will hail with delight the prospect of their elected representatives being able to control the useless and perfectly avoidable noises which have so long tormented them. It is not, however, as a measure against noise alone that this decision is of the greatest importance. What it declares is not only that a representative body may frame bye-laws restricting the liberty of action of persons who come under their operation, or restraining them from acts which but for such bye-laws they would be at liberty to do or not do as they pleased; but that a body making such bye-laws may employ its own police to put them in force—a matter which practically is of even greater importance.

To the private individual, this is a point of the extremest interest. For the householder to have to order an organ-grinder to "move on," to have to give his reason, and to have to call a constable, has been, as we all know, sufficient to render the law in regard to organ-grinding practically a dead letter.

The power to enforce bye-laws given to the police puts the care of the streets on the proper shoulders, andmay have far-reaching consequences, for there are matters of much greater importance than street "music" which can be effectually dealt with by local authorities if only they may use their own officers to put their rules in force. It has been the necessity of proving nuisance, or annoyance, or individual damage that has left the citizen at the mercy of those who use the streets for their own purposes. Once allow the police to enforce bye-laws regulating conduct in the streets, and to summons those who sin against them, and we see many openings for a sanitarily-minded County Council.

